# The peritumoral edema index and related mechanisms influence the prognosis of GBM patients

**DOI:** 10.3389/fonc.2024.1417208

**Published:** 2024-10-29

**Authors:** Zhansheng Fang, Ting Shu, Pengxiang Luo, Yiqing Shao, Li Lin, Zewei Tu, Xingen Zhu, Lei Wu

**Affiliations:** ^1^ Department of Neurosurgery, The 2nd Affiliated Hospital, Jiangxi Medical College, Nanchang University, Nanchang, China; ^2^ Jiangxi Key Laboratory of Neurological Tumors and Cerebrovascular Diseases, Nanchang University, Nanchang, China; ^3^ Jiangxi Health Commission Key Laboratory of Neurological Medicine, Nanchang University, Nanchang, China; ^4^ Institute of Neuroscience, Nanchang University, Nanchang, China; ^5^ Department of Medical Imaging Center, The 2nd Affiliated Hospital, Jiangxi Medical College, Nanchang University, Nanchang, China

**Keywords:** glioblastoma, peritumoral brain edema, MRI, bioinformatics glioblastoma, bioinformatics, image segmentation

## Abstract

**Background:**

Peritumoral brain edema (PTBE) represents a characteristic phenotype of intracranial gliomas. However, there is a lack of consensus regarding the prognosis and mechanism of PTBE. In this study, clinical imaging data, along with publicly available imaging data, were utilized to assess the prognosis of PTBE in glioblastoma (GBM) patients, and the associated mechanisms were preliminarily analyzed.

**Methods:**

We investigated relevant imaging features, including edema, in GBM patients using ITK-SNAP imaging segmentation software. Risk factors affecting progression-free survival (PFS) and overall survival (OS) were assessed using a Cox proportional hazard regression model. In addition, the impact of PTBE on PFS and OS was analyzed in clinical GBM patients using the Kaplan–Meier survival analysis method, and the results further validated by combining data from The Cancer Imaging Archive (TCIA) and The Cancer Genome Atlas (TCGA). Finally, functional enrichment analysis based on TCIA and TCGA datasets identified several pathways potentially involved in the mechanism of edema formation.

**Results:**

This study included a total of 32 clinical GBM patients and 132 GBM patients from public databases. Univariate and multivariate analyses indicated that age and edema index (EI) are independent risk factors for PFS, but not for OS. Kaplan–Meier curves revealed consistent survival analysis results between IE groups among both clinical patients and TCIA and TCGA patients, suggesting a significant effect of PTBE on PFS but not on OS. Furthermore, functional enrichment analysis predicted the involvement of several pathways related mainly to cellular bioenergetics and vasculogenic processes in the mechanism of PTBE formation. While these novel results warrant confirmation in a larger patient cohort, they support good prognostic value for PTBE assessment in GBM.

**Conclusions:**

Our results indicate that a low EI positively impacts disease control in GBM patients, but this does not entirely translate into an improvement in OS. Multiple genes, signaling pathways, and biological processes may contribute to the formation of peritumoral edema in GBM through cytotoxic and vascular mechanisms.

## 1 Introduction

Glioblastoma (GBM) is one of the most lethal and intractable solid tumors, accounting for 16% of all primary central nervous system (CNS) tumors and 81% of all CNS malignancies (1, 2). Despite combination therapy involving surgery, radiotherapy, chemotherapy, immunotherapy, and targeted therapy, the median survival of GBM patients remains only 12–14 months, whereas three- and five-year survival rates stand at 16% and 9.8%, respectively (3–5).

The mechanisms underlying peritumoral brain edema (PTBE), a characteristic imaging manifestation of intracranial gliomas, remains to be fully elucidated. Vascular and lymphatic dissemination represent typical metastatic modes of peripheral malignant tumors. However, glioma may metastasize either through direct infiltration into the surrounding normal tissue or via the cerebrospinal fluid (CSF). Direct invasion may alter the physiological activity and microenvironment of the surrounding normal brain cells, eventually leading to the formation of an edematous zone around the tumor (6). GBM is characterized by invasive growth, and typically shows no discernible boundaries with the surrounding normal brain tissue. In the PTBE region is difficult to accurately distinguish tumor boundary, and the presence of scattered tumor cells increases the possibility of tumor recurrence after operation (6–8). While the use of CT, MRI, and other imaging methods for studying tumor morphology in relation to tumor prognosis has been long reported, a unified morphological evaluation index for measuring brain tumor size and edema degree is still lacking. Evaluation methods for tumor and edema degree encompass volume formula methods such as $\frac{2}{3}Sh$ and $\frac{1}{2}ABC$ (9), and indirect evaluations using maximum diameter, width, and two-dimensional area (6). This variety in methods contributes to a lack of unity and scientific rigor in the approaches applied. Moreover, the volume estimation method is only applicable to occupying lesions with relatively regular volumes and lacks sufficient accuracy for irregular brain tumors and edema. Berntsen et al. (10) conducted a comparison of tumor volumes using progressive 3D volume segmentation and 2D volume estimation. They found that volumetric calculations outperformed 2D estimates and were less likely to overlook actual tumor progression. Moreover, larger tumors, even those with mild edema suggested at the 2D level, may be larger when calculated in 3D. Even if PTBE is evident in small tumors, calculations may indicate small edema. Therefore, predicting prognosis based on estimated edema volume and maximum cross-sectional accumulation lacks rigor and overlooks the causal relationship between the tumor and the edema.

In this study, with the assistance of two senior imaging and neurosurgery professionals, we aimed to accurately quantify tumor volume, edema volume, enhanced volume, and necrosis volume from MRI sequences using ITK-SNAP, a semi-automated segmentation software. Further, the prognostic impact of PTBE on progression-free survival (PFS) and overall survival (OS) was assessed by evaluating the ratio of edema volume to tumor volume in GBM imaging in combination with clinical data. Results were externally validated using GBM patient data in The Cancer Imaging Archive (TCIA) and The Cancer Genome Atlas (TCGA) databases. Complex tumor genetic information can be addressed by high-throughput data processing, which enables the discovery of associations between relevant genes and signaling pathways and allows inferring tumor prognosis (11, 12). We thus used bioinformatics to perform, based on calculated edema index (EI) scores, differential gene expression analysis in TCGA patients, and conducted Gene Ontology (GO) and Kyoto Encyclopedia of Genes and Genomes (KEGG) analyses to illuminate possible molecular mechanisms associated with the formation of PTBE.

## 2 Materials and methods

### 2.1 Study subjects

This retrospective study utilized two main sources of imaging data from study participants. The first source encompassed clinical patient imaging and clinical data from July 1, 2019, to July 1, 2021, collected at Nanchang University Hospital No. 2 for neurosurgical treatment with a pathologic diagnosis of GBM. The second source included imaging and survival data from GBM patients in TCIA Oncology Archive (https://www.cancerimagingarchive.net/) and TCGA database (https://portal.gdc.cancer.gov/) for validation. The main endpoints in this study were OS and PFS (13).

### 2.2 Inclusion and exclusion criteria for patients

The inclusion criteria for patients in this study were as follows: (i) The pathological grades of the resected tissues, examined under the microscope, conformed to the IV grade of GBM as per the 2016 edition of WHO CNS Tumor Classification; (ii) Complete perioperative imaging data and medical records of patients could be obtained from the medical record system, along with a complete follow-up process and imaging data from the datacenter of the second affiliated Hospital of Nanchang University; (iii) Prior to the craniocerebral MRI examination, the patients had not received any special treatments such as surgery, radiotherapy/chemotherapy, or immunotherapy. The exclusion criteria were as follows: (i) lack of complete clinical and perioperative data and no complete follow-up process, which precluded determination of the survival status of postoperative patients; (ii) no obvious enhancement lesions observed in preoperative craniocerebral MRI, and cases where combined with other related imaging sequences, the boundary of tumor and edema could not be clearly judged; (iii) the patient had a history of other craniocerebral operations, severe traumatic brain injury, and/or multiple intracranial gliomas; (iv) patients who had other conditions that may affect their survival in the short term.

### 2.3 Imaging methods

All GBM patients admitted to our hospital underwent an MRI examination using a Siemens MRI scanner with a magnetic field strength of either 1.5T or 3.0T. An intravenous injection of 0.1 mmol/kg of Magnevist, an enhanced scanning contrast agent, was administered (14). Imaging sequences included T1-weighted imaging (T1WI), T2-weighted imaging (T2WI), T2-Fluid attenuated invasion recovery (T2-FLAIR), and T1WI contrast-enhanced (T1+C). The image storage and communication systems of the hospital’s imaging center were utilized to gather DICOM images of all GBM patients included in this study.

### 2.4 Imaging analysis

**Figure 1:**
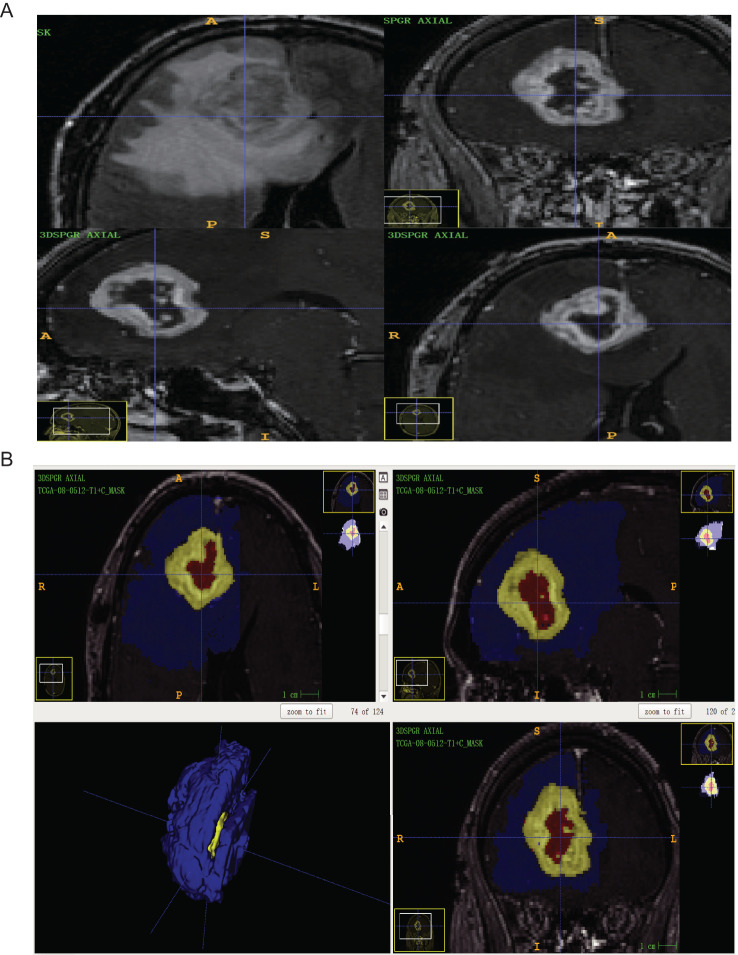
**(A)** The GBM patients with routine MRI scan (ID: TCGA-08-0512) were T2-FLAIR axial sequence and T1 + C three-plane, respectively; **(B)** ITK-snap software mapped the ROI of GBM patients with axial, sagittal, coronal, and 3D images (Blue is edema volume, yellow is tumor enhancement volume and red is tumor necrosis volume).

Under the supervision of senior neurosurgeons, ITK-SNAP (4.1.0) software was utilized to outline the region of interest in all GBM cases included in this study. T1WI, T2WI, T2-FLAIR, and T1+C scan sequences were used to determine preoperative PTBE, tumor site, and contrast-enhanced tumor volume. Tumor volume was calculated using the T1+C sequence, while edema volume, which includes tumor volume, was calculated using T2WI or T2-FLAIR. PTBE involves mainly white matter surrounding the tumor, and shows a low signal in the T1WI sequence and a high signal in T2WI or T2-FLAIR sequence images. Therefore, in this study, we took the difference between T1+C and T2WI or T2-FLAIR as the PTBE volume (**Figure 1**). Cysts are defined as circular regions that match the CSF signal, showing a low T1WI signal and high T2WI signal as well as a smooth, regular, slightly enhanced thin wall (15). In this study, the peritumoral EI and contrast-enhanced tumor ratio (CTR) were utilized as reference indices to quantify the volume of each component and evaluate the degree of PTBE and the volume of the enhanced tumor, respectively. The following formulas were applied $EI=\frac{V_{\text{tumor}}+V_{\text{edema}}}{V_{\text{tumor}}}$ (16); $CTR=\frac{V_{\text{enhanced}}}{V_{\text{tumor}}}$ (17).

### 2.5 Survival analysis

We evaluated the impact of clinical variables and imaging features on postoperative OS and PFS in patients with GBM using Cox proportional hazard regression analysis. Initially, X-tile software (https://medicine.yale.edu/lab/rimm/research/software/) was utilized to calculate the cutoff values of continuous variables such as age, EI, and CTR, which were then categorized into different groups. Next, a semi-parametric regression model (Cox proportional hazard regression analysis) was utilized to evaluate the risk factors related to PFS and OS in GBM patients by estimating hazard ratio (HR), 95% confidence interval (95% CI), and P value, among other measures. Finally, based on the collected clinical data, Kaplan–Meier analysis was used to visually represent OS and PFS survival curves related to PTBE and to analyze the effect of EI on survival and prognosis.

### 2.6 Verification of patient survival analysis based on TCGA database

TCIA (https://www.tcia.at/home), the official image repository of the National Cancer Institute, contains radiological or pathological annotations, image classification and segmentation data, radiological features, and derived or reprocessed images (18). TCIA is an imaging repository based on TCGA database, through which clinical and gene expression profiles can be collected through corresponding ID numbers (19). This information in the TCGA database was utilized to verify the impact of EI on PFS and OS of GBM patients.

### 2.7 Identification of differentially expressed genes and functional enrichment analyses

The TCGA and GEO (https://www.ncbi.nlm.nih.gov/geo/) databases were combined to screen out differentially expressed genes (DEGs) related to EI. The corresponding gene expression dataset for GBM patients was obtained from TCIA, and the “limma” package of R software (https://www.r-project.org/) was utilized to analyze gene expression differences. The patients were categorized into groups based on degree of edema (EI). Differential expression analysis was conducted to identify DEGs related to edema, and a volcano map was subsequently drawn. Using the DAVID bioinformatics database, GO and KEGG pathway enrichment analyses were performed using the R package “cluster Profiler.” *p* < 0.05 was considered significant.

### 2.8 Statistical analyses

All the data collected from available clinical records and TCGA were processed using Statistical Package for Social Sciences, and gene expression differences were analyzed using R language. Values of normally distributed data were expressed as mean ± SD (standard deviation), while values of non-normally distributed data were expressed as median and interquartile range. Categorical variables were expressed as absolute values. *p* < 0.05 was considered significant.

## 3 Results

### 3.1 Patient characteristics

Following a comprehensive screening, 32 clinical GBM patients were selected. Their general information was sourced from the electronic medical record system in our hospital (**Table 1**). The average age of the patients was 58 years, with a male to female ratio of 1.7:1 (20:12). Tumors with cystic degeneration and invasion of the lateral ventricle accounted for 21.9% and 28.1% of cases, respectively. Tumors with single-lobe involvement accounted for 62.5% of cases. The average volumes of the tumor, edema, and contrast-enhanced tumor, measured using ITK-SNAP software, were 48.04 ± 30.57 cm³, 90.10 ± 53.87 cm³, and 31.36 ± 18.34 cm³, respectively.

**Table 1:** Baseline information for clinical patients.

Clinical Characteristics		(mean SD/med(IQR))/n(%)
Age		57.94 ± 10.00
Sex	Male	20 (62.5%)
	Female	12 (37.5%)
EI		3.38 ± 1.73
Tumor volume(cm^3^)		48.04 ± 30.57
Edema volume(cm^3^)		90.10 ± 53.87
Contrast-enhanced Tumor volume(cm^3^)		31.36 ± 18.34
Necrotic tumor volume(cm^3^)		8.91 (3.59~15.74)
CTR		0.79 (0.62~0.86)
cystic change	YES	7 (21.9%)
	NO	25 (78.1%)
Postoperative treatment	YES	15 (46.9%)
	NO	17 (53.1%)
Basic diseases(Hypertension/Diabetes)	YES	3 (8.4%)
	NO	29 (90.6%)
Edema involving lateral ventricles	YES	9 (28.1%)
	NO	23 (71.9%)
Number of lobes involved	single	20 (62.5%)
	multiple	12 (37.5%)

SD, Standard deviation; IQR, Interquartile range; EI, Edema index; CTR, Contrast-enhanced Tumor Ratio.

### 3.2 Analysis of survival predictors for GBM

We evaluated the impact of age, EI, and CTR on survival in GBM patients. For these continuous variables, the best cutoff values calculated by X-tile (3.6.1) software were 60 years, 3.5, and 0.8, respectively (**Table 2**). A univariate regression analysis of OS revealed that gender (HR: 2.624, 95% CI: 1.124–6.142, *p* = 0.026), age (HR: 2.628, 95% CI: 1.202–5.000, *p* = 0.015), CTR (HR: 2.382, 95% CI: 1.080–5.000, *p* = 0.032), and cystic change (HR: 0.188, 95% CI: 0.055–0.639, *p* = 0.007) were risk factors. No independent risk factors were found in the multivariate Cox model. In addition, postoperative PFS single-factor regression analysis revealed that gender (HR: 3.759, 95% CI: 1.577–9.148, *p* = 0.004), age (HR: 3.173, 95% CI: 1.347–7.474, *p* = 0.008), EI (HR: 2.920, 95% CI: 1.290–6.611, *p* = 0.01), and cystic changes (HR: 0.251, 95% CI: 0.0992, *p* = 0.008) were risk factors. In turn, multivariate Cox models showed that age (HR: 3.423, 95% CI: 1.295–9.046, *p* = 0.013) and EI (HR: 2.771, 95% CI: 1.157–6.635, *p* = 0.022) were independent risk factors for postoperative PFS in GBM patients. These results are summarized in **Table 3**.

**Table 2:** The cut-off value of continuous variables.

variate	Cut-off value
Age(yeas)	60
EI	3.5
CTR	0.8

**Table 3:** Cox analysis of OS and PFS in clinical GBM patients.

Factors	OS						PFS					
	Univariate analysis		P	Multivariate analysis		P	Univariate analysis		P	Multivariate analysis		P
	HR	95% CI		HR	95% CI		HR	95% CI		HR	95% CI	
Gender	2.624	1.124-6.142	0.026*	1.539	0.549-4.316	0.413	3.759	1.544-9.148	0.004*	2.409	0.875-6.637	0.089
Age	2.628	1.202-5.744	0.015*	2.375	0.882-6.398	0.087	3.173	1.347-7.474	0.008*	3.423	1.295-9.046	0.013*
EI	2.123	0.955-4.717	0.065	-	-	-	2.920	1.290-6.611	0.01*	2.771	1.157-6.635	0.022*
CTR	2.382	1.080-5.253	0.032*	1.019	0.330-3.142	0.974	2.093	0.980-4.469	0.056	-	-	-
Cystic change	0.188	0.055-0.639	0.007*	0.260	0.053-1.279	0.098	0.251	0.091-0.692	0.008*	0.413	0.128-1.337	0.140
Radio/chemotherapy	0.871	0.409-1.858	0.721	-	-	-	0.670	0.326-1.379	0.277	-	-	-
Involving lateral ventricle	1.045	0.457-2.389	0.918	-	-	-	1.081	0.478-2.442	0.852	-	-	-
Hypertension/Diabetes	0.858	0.400-1.841	0.695	-	-	-	1.282	0.692-2.377	0.430	-	-	-
Hypertension/Diabetes	1.442	0.430-4.839	0.554	-	-	-	1.527	0.447-5.214	0.499	-	-	-

*P<0.05.

### 3.3 Kaplan–Meier survival analysis and validation of prognostic EI

**Figure 2:**
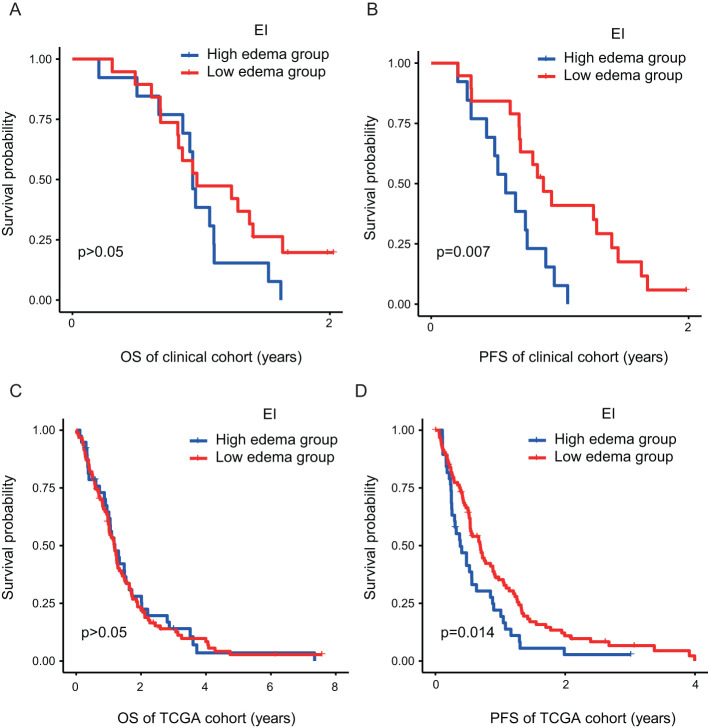
The PTBE of GBM patients has a significant effect on PFS **(B–D)**, but no significant effect on OS **(A–C)**. EI, Edema Index; H, Hight degree edema; L, Low degree edema.

The analysis of clinical samples in our internal GBM experimental cohort (n = 32) indicated a median PFS of 211 days (95% CI: 141.715–280.285) for the high-edema group and 318 days (95% CI: 247.074–388.926) for the low-edema group *(p* < 0.05) (**Figure 2B**). In view of the insufficient sample size of our patient cohort, patient data in the TCIA/TCGA databases were analyzed using Kaplan–Meier analysis to further confirm the impact of EI on survival and prognosis. Results showed that the median PFS was 175 days (95% CI:113.067–236.933) in the high-edema group and 253 days (95% CI:194.727–311.273) in the low-edema group (*p* < 0.05) (**Figure 2D**). We found no significant difference for EI regarding OS among all studied patients. The results of the two cohorts were thus consistent, suggesting that EI had a significant effect on PFS, but not on OS, in GBM patients with PTBE (**Figures 2A, C**).

### 3.4 Biological processes and pathways associated with PTBE

**Figure 3:**
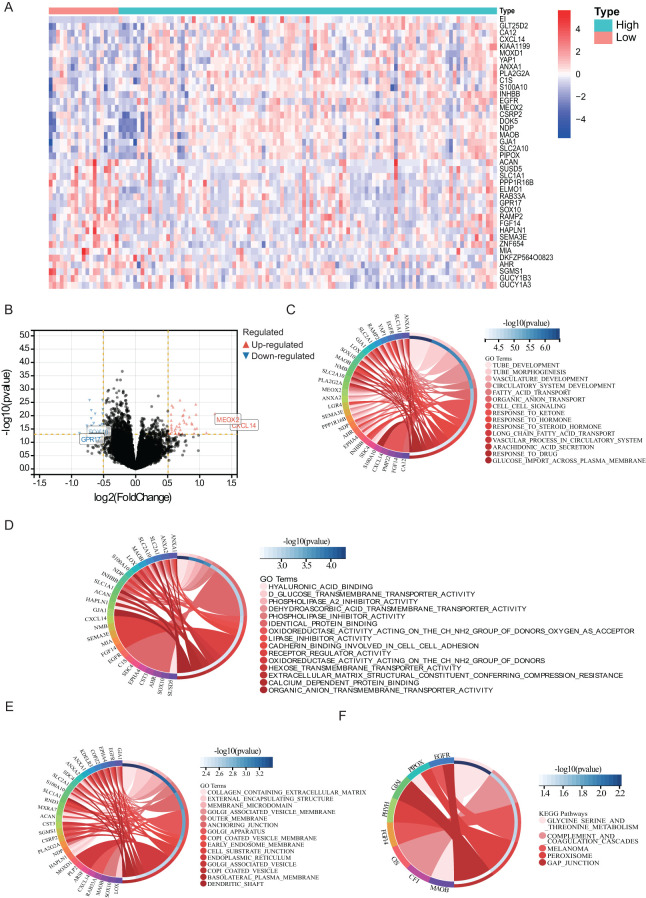
Biological processes and pathways associated with PTBE. The relationship **(A)** between the aberrant expression of DEGs **(B)** and PTBE of gliomas in the TCGA datasets. Functional annotations and predicting signaling pathways: Biological process **(C)**, molecular function **(D)**, cell component **(E)** and KEGG pathway **(F)** enrichment.

Differential expression analysis was performed using the limma package in R language, applying a threshold of log2FC > 0.5, *p* < 0.05. A heat map of the results (**Figure 3A**) revealed significant correlations with edema extent for 148 DEGs. Of these, 47 showed a positive correlation and 101 a negative correlation (**Figure 3B** **).** The DEGs with the strongest correlations included MEOX2, CXCL14, GPR17, and SOX10. In addition, we surveyed biological processes and pathways potentially involved in the formation of edema through GO and KEGG functional enrichment analysis. GO analysis of the 148 DEGs showed enrichment in biological processes including tube development and morphogenesis, vasculature and circulatory system development, fatty acid and organic anion transport, cell–cell signaling, response to ketone, hormone, drug, and steroid hormone, vascular process in circulatory system, arachidonic acid secretion, and glucose import across plasma membrane (**Figure 3C**). Molecular function terms like sodium hyaluronate, redox reactions, lipase and phospholipase activities, glucose transport, and organic anion transport after intercellular adhesion were identified as also enriched in these DEGs (**Figure 3D**). Cellular component (CC) analysis highlighted collagen-containing extracellular matrix, lipid membrane, and Golgi-associated vesicle as the main DEG-enriched terms (**Figure 3E**). In turn, KEGG pathway analysis revealed that the DEGs were mostly enriched in glycine, serine and threonine metabolism, complement and coagulation cascades, melanoma, peroxisome, and gap junction (**Figure 3F**).

### 3.5 Gene set enrichment analysis of edema-related signaling pathways in GBM

**Figure 4:**
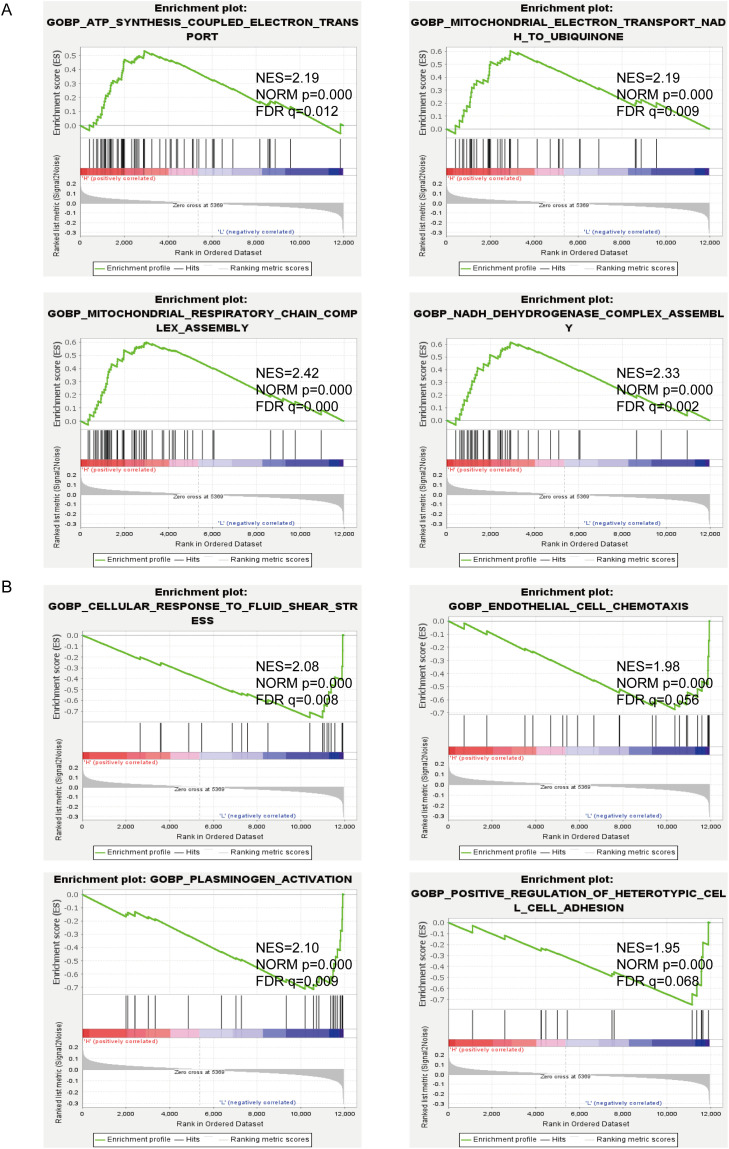
GSEA enrichment between high **(A)** and low **(B)** edema groups.

To investigate potentially relevant signaling pathways in PTBE, we performed gene set enrichment analysis (GSEA) comparing the high- and low-edema risk groups. Our results showed that ATP synthesis coupled electron transport, electron transport from NADH to ubiquitin in mitochondria, mitochondrial respiratory chain complex assembly, and NADH dehydrogenase complex assembly, among other terms, were differentially enriched in the high-EI phenotypes (**Figure 4A**). In contrast, cellular response to laminar fluid shear stress, endothelial cell chemotaxis, and positive regulation of heterotypic cell–cell adhesion were enriched in the low-EI GBM group (**Figure 4B**). These results provide insights into the cellular processes related to PTBE.

## 4 Discussion

GBM represents the most common primary intracranial malignant tumor, characterized by high mortality, high recurrence rate, and poor prognosis. As GBM poses a significant threat to global public health, international efforts are devoted to test and develop new treatment methods, drugs, and advanced surgical techniques (20, 21). However, current treatment methods for GBM, including mainly surgical resection supplemented by radiotherapy, chemotherapy, and molecular immunotherapy, show clear limitations and the prognosis of GBM patients remains poor. Full use by neurosurgeons of conventional imaging examinations to directly or indirectly evaluate the prognosis of glioma patients, and even predict gene mutations, will be of great significance for the treatment of patients (22, 23). MRI is recognized as a non-invasive technique with massive potential. It furnishes meaningful parameter information in the preoperative diagnosis and staging of glioma as it allows accurate assessment of tumor size, tumor location, surrounding anatomical structures, local metabolism, and anatomical relationship with functional areas.

PTBE is a typical indicator of GBM in the MRI scan, manifested as high signal intensity on T2WI or T2-FLAIR. Severe PTBE can cause cerebral herniation due to intracranial hypertension, resulting in neurological impairment. Current studies lack a unified standard and clear conclusion on how to evaluate the degree of PTBE. This could be attributed to the common growth pattern of GBM, along the direction of the white matter, resulting in various tumor morphologies (24). Therefore, 3D MRI measurements of craniocerebral tumors may be more informative than 2D horizontal or vertical measurements (25–27). In this study, EI based on 3D segmentation was used as an indicator to evaluate the degree of PTBE, and the effects of PTBE on OS and PFS of GBM patients were analyzed. Unlike previous EI estimations, which excluded tumor volume, the ratio of edema volume to tumor volume, which emphasizes a causal relationship and reflects more objectively the impact of edema on patient prognosis, was used here as an evaluation index.

3D imaging metrics such as tumor volume and enhanced volume have been described as potential predictors of efficacy response and survival in the focal treatment of extracranial and intracranial malignancies (28, 29). However, PTBE as a significant factor for the high recurrence rate and high mortality of GBM, and its prognostic value, remain subjects of controversy (30–32). For this reason, we dismissed 2D data usually screened in clinical practice and used instead ITK-SNAP software for 3D reconstruction of the tumor; this allowed us to collect accurate 3D data to further analyze the impact of edema on the survival of GMB patients. Using a simple measure to distinguish the degree of edema (i.e., the distance between the edge of the enhanced tumor and the edge of the edema), Schoenegger et al. (33) showed that extensive PTBE in preoperative MRI can lead to severe nerve damage and predicts worse survival in GBM. Their survival analysis results were consistent with the conclusions of Wu et al. (30), suggesting that PTBE is a poor prognostic factor for GBM patients. However, Henker et al. (34) and Auer et al. (17) suggested that PTBE is not an imaging feature predictive of OS in GBM, proposing instead CTR as an independent risk factor for OS prognosis. Our results also show that in GBM patients EI is not an independent risk factor for OS, but it is instead for PFS. In the evaluation of tumor treatment efficacy, PFS and OS are two key indicators. PFS measures the time during and after treatment in which the disease does not worsen, reflecting the stage of disease control (35). OS refers to the total survival time of the patient. This study demonstrates that although a low EI is associated with better disease control than a high EI in GBM patients, this benefit does not fully translate into an improvement in OS.

GBM is well-known for its high aggressiveness, which may reduce the diagnosable local tumor mass, particularly in functional areas of the CNS (36). PTBE is not merely a reactive edema. It is also one of the markers of GBM’s high invasiveness, denoting the spreading of tumor cells to the surrounding edematous area (7). Even if gliomas are completely removed by surgical resection, most GBM patients are still likely to relapse. In this regard, it was reported that including the peritumoral edema in the radiation target area can significantly reduce the rate of marginal recurrence (37). Furthermore, research has confirmed that supramaximal resection (SMR), including perilesional edema, helps improve PFS and OS in patients with grade 4 astrocytoma, both IDH wild-type and IDH mutant (38). This implies that a lower degree of edema, which suggests reduced spread of tumor cells, is more beneficial for controlling GBM progression. This is also why PFS is meaningful, while OS is not. Consequently, a more aggressive surgical approach could potentially lead to improved clinical outcomes.

GBM is an age-related neurological disease, and its treatment efficacy and prognosis decline with age (39). In this study, both PFS and OS estimates showed that the prognosis of GBM patients <60 years old was significantly better than that of patients ≥60 years old (*p* < 0.05), which means that older age is associated with worse prognosis. A dynamic interaction was described between the aging brain, the immune system, and GBM (40, 41). Gender is an intriguing variable in GBM research. Some studies showed that compared with male patients, female patients had significantly better prognosis, with five-year survival rates of 8.3% compared to 6.8% in male patients (42, 43). In turn, our Cox univariate analysis showed that gender was a risk factor for PFS and OS in GBM patients, but we found no significant difference in Cox multivariate analysis. A better prognosis in women than in men may be consequence of protective effects of estrogen acting on glioma cells (44). It is also well recognized that differences in sex hormones can affect the immune response (45). Cystic degeneration can be distinguished from tumor necrosis on MRI scanning, and frequently shows smooth, thin, distinctly enhanced edges, with high T2 signal indicating the presence of cystic fluid. ​In this study, 21.9% of GBM patients had significant cystic lesions, consistent with the 7%–23% cystic rate reported by Lee et al. (46).

We analyzed the potential mechanisms underlying PTBE formation by searching for DEGs associated with peritumoral edema in GBM cases screened from TCIA and TCGA databases. Among a total of 148 DEGs thus identified, MEOX2, CXCL14, GPR17, and SOX10 were the most significant. MEOX2 is a homeobox gene that suppresses the growth of endothelial and vascular smooth muscle cells and stimulates cell proliferation and motility (47). The chemokine CXCL14 modulates GBM-associated stromal cells, modifies the immune microenvironment, and enhances the invasiveness of glioma cells (48, 49). SOX10 and GPR17 were primarily expressed in glioma cells from the low-edema group of patients. In past studies, their expression was correlated with inhibition of glioma cell proliferation and invasion (50, 51). However, whether SOX10 and GPR17 directly influence PTBE requires further investigation. GO and KEGG analyses suggested that PTBE-related DEGs closely influenced various tissue structures, processes, and signaling pathways, including extracellular matrix, microvessel formation, energy metabolism, cell–cell or plasma membrane transport systems (including organic anions and hexose), complement and coagulation cascades, and cell adhesion. The main cellular components of the blood–brain barrier (BBB) are endothelial cells with tight and adherens junctional protein complexes. This specialized vascular endothelium, together with astrocytes, and pericytes attached to the basement membrane, determines a highly selective permeability for the passage of ions and macromolecules from the extracerebral environment (52).

Malignant GBM requires a robust vascular system to supply the growing nutritional and metabolic demands of the tumor. Vascular channels with endothelium-like characteristics are formed in GBM tissues, in a process known as vasculogenic mimicry (53) (54). Furthermore, significant remodeling of the extracellular matrix, evidenced by increased deposition of fibrillary proteins (collagen, laminin, fibronectin) and upregulation of specific glycoproteins (tenascins), proteoglycans (chondroitin sulfates, versican, syndecan), focal adhesion proteins (FAK, vinculin), and degradative enzymes (MMPs) facilitate glioma cell migration and invasion (55). This leads to diffusion of macromolecules and water into the brain parenchyma, resulting in PTBE formation (56, 57).

GSEA indicated that PTBE may be influenced by altered cellular bioenergetics, in association with changes in mitochondrial respiratory chain activity, and by vascular changes involving laminar fluid shear stress, endothelial cell chemotaxis, and positive regulation of heterotypic cell–cell adhesion. In addition, extracellular matrix and oxidoreductase activity, among other signaling pathways, showed an association with PTBE formation according to our GSEA results. Thus, we hypothesize that the above processes and their associated signaling pathways play a critical role in the progression of PTBE in gliomas.

We analyzed the association of quantitative MRI features with survival of GBM patients and further verified the prognostic value of PTBE on OS and PFS in TCGA and TCIA GMB cohorts. The results showed that a low EI has positive implications for short-term survival, but this does not obviously translate into OS benefits. This is possibly related to differential gene expression, alterations in signaling pathways, and other biological effects collectively contributing to PTBE formation. Due to ethical limitations, and to avoid additional damage, we were not able to obtain PTBE tissue from GBM patients. There is thus a need to verify these findings through molecular studies.

In our retrospective study of clinical cases, we encountered several key challenges. First, this study is centered on the analysis of a subset of GBM patients whose preoperative brain MRI distinctly delineates the boundary between the tumor and edema. However, this significantly limits the sample size of the experimental group, thereby making it challenging to rule out potential research biases. In addition, the compression effect of the tumor can cause angioedema, which can be confused with edema caused from aggressive GBM, as both types of edema show high signal on T2WI/FLAIR images (58). Therefore, in subsequent studies it is recommended to use deep learning techniques to distinguish angioedema from infiltrative edema to further improve the accuracy of imaging results. Nonetheless, the significance of this study lies in its potential to positively influence the treatment and prognosis of these specific patients. While these findings may not be applicable to all GBM patients, we believe that they hold substantial significance for a better understanding of GBM and may pave the way for personalized treatments. Second, with the continuous updating of the classification standards for GBM, the GBM classification framework based on 2016 is no longer suitable for current research needs. Hence, our ongoing research focuses on whether PTBE can reveal new findings in different GBM subtypes. Lastly, our bioinformatics analysis relied solely on the two public databases, TCGA and TCIA. Although they provide valuable data resources, the depth of analysis needs to be improved and experimental verification is lacking, which weakens to some extent the reliability and extensibility of our research conclusions. To overcome these limitations, we plan to further explore more diverse data sources and experimental methods to ensure comprehensiveness and accuracy of our findings. For instance, multi-center clinical studies with large sample size will help reduce potentially complex biases. In turn, advanced deep learning algorithms combined with multi-modal imaging techniques can be utilized to distinguish different types of edema. Based on the latest GBM classification standards, genomic and molecular profiling techniques can be employed to classify GBM more accurately and identify potential biomarkers associated with PTBE. Finally, *in vitro* and *in vivo* animal models can be used to model and validate clinical features of GBM development and progression. Implementing these strategies would strongly minimize current limitations and enhance the robustness and accuracy of studies on GBM and associated PTBE. All in all, Evolving as a critical indicator in glioblastoma treatment, Edema Index (EI) facilitates the assessment of peritumoral brain edema (PTBE) extent. Surgical planning is enhanced by delineating edematous regions necessitating resection or targeted therapy. Quantification of PTBE via EI aids in evaluating tumor aggressiveness and prognosticating patient outcomes. Integrating EI into routine clinical practice enhances the precision of glioblastoma management, ultimately improving patient care.

## Data Availability

The datasets presented in this study can be found in online repositories. The names of the repository/repositories and accession number(s) can be found in the article/supplementary material.
